# A network meta-analysis evaluating the efficacy and safety of adjuvant therapy after nephrectomy in renal cell carcinoma

**DOI:** 10.1186/s12894-024-01441-8

**Published:** 2024-03-07

**Authors:** Lingyu Guo, Tian An, Zhixin Huang, Tie Chong

**Affiliations:** 1 Department of Urology, The Second Affiliated Hospital of Xi’an Jiaotong University, 157 West Fifth Road, Xi’an, 710000 China; 2 Department of Dermatology and Plastic Surgery, The Second Affiliated Hospital of Shaanxi University of Traditional Chinese Medicine, Xianyang, China

**Keywords:** Renal cell carcinoma, Network meta-analysis, Adjuvant therapy, Efficacy

## Abstract

**Background:**

In the past few years, there has been a continuous rise in the occurrence of renal cell carcinoma (RCC), with RCC recurrence becoming the primary factor behind fatalities. Despite numerous clinical trials, the impact of different medications on the long-term survival of patients with RCC after surgery remains uncertain. This network meta-analysis aimed to evaluate the impact of various medications on the survival and safety of drugs in individuals with RCC following nephrectomy.

**Methods:**

We conducted a thorough search in various databases, including CNKI, WAN FANG DATA, VIP, Web of Science, Cochrane Library (CENTRAL), PubMed, Scopus, and Embase, for articles published prior to June 2, 2023. This meta-analysis incorporated randomized controlled trials (RCTs).

**Results:**

The analysis included 17 studies with 14,298 participants. The findings from the disease-free survival (DFS) analysis indicated that pembrolizumab demonstrated efficacy in enhancing DFS among patients with RCC following nephrectomy when compared to the placebo group (HR = 0.83, 95%CI 0.70 to 0.99). None of the drugs included in the study significantly improved overall survival (OS) and recurrence-free survival (RFS) after nephrectomy. For adverse events (AEs), sorafenib, pazopanib, sunitinib, and nivolumab plus ipilimumab interventions showed a higher incidence of adverse events compared with placebo.

**Conclusion:**

The network meta-analysis yielded strong evidence indicating that pembrolizumab could potentially enhance DFS in patients with RCC following nephrectomy, surpassing the effectiveness of a placebo.

**Supplementary Information:**

The online version contains supplementary material available at 10.1186/s12894-024-01441-8.

## Introduction

The urinary system is commonly affected by renal cell carcinoma (RCC), a widespread malignant tumor. The locoregional disease is diagnosed in approximately 80% of patients [1]. Nevertheless, even after going through a surgical procedure, numerous individuals encounter a relapse, with recurrence rates over a span of 5 years varying from 10% in patients with low risk to as high as 68% in patients with high risk [2, 3]. Either partial or radical nephrectomy is the typical approach for treating locoregional RCC. Although numerous advancements have been in treating advanced illnesses in recent years, the effectiveness of post-nephrectomy adjuvant therapy remains unclear.

In patients with advanced disease, sunitinib and sorafenib, which are Vascular endothelial growth factor receptor (VEGFR) inhibitors, have demonstrated the ability to improve progression-free survival. Indeed, the prolonged utilization of these inhibitors in advanced stages has been noted to enhance the median overall survival period from 13 months to more than 29 months [4].

The probability of recurrence within the initial five years after the procedure was highest among patients who were considered free of disease following nephrectomy for RCC. Nearly all T4 patients experience relapse after nephrectomy, approximately 50% of T3 patients, and up to 26% of T2 patients, making the primary tumor stage a widely recognized prognostic indicator [5]. Increased tumor nuclear grade and the existence of sarcomatoid characteristics have also been recognized as separate variables linked to an elevated possibility of disease recurrence [6]. Patients with resectable soft tissue metastases at the time of diagnosis and primary kidney tumors (stage M1 disease) constitute another category of individuals who could potentially gain advantages from adjuvant therapy [7]. Even after successful removal of the kidney and complete removal of metastatic growths, individuals who are eligible for surgery still face a significant chance of experiencing a relapse and mortality within a span of 5 years post-operation, without any existing options for additional treatment.

In the United States of America (USA), the adjuvant therapy approval for patients at high risk of recurrence was granted to sunitinib, an anti-VEGF tyrosine kinase inhibitor(TKI), due to the S-TRAC trial’s results showing enhanced disease-free survival (DFS) in comparison to a placebo [8]. Nevertheless, this study did not demonstrate any significant benefit in terms of overall survival. Additional tests on anti-VEGF medications like pazopanib, axitinib, and sorafenib have failed to achieve their main effectiveness goals. At the same time, sunitinib has yielded inconsistent outcomes regarding DFS in randomized phase 3 trials [9–11]. Although sunitinib is not universally endorsed as a treatment in this context, there is insufficient strong evidence supporting its effectiveness for RCC.

The emergence of immune checkpoint inhibitors provides a new treatment option for tumor patients. Some researchers have found that nivolumab combined with ipilimumab can significantly improve the prognosis of RCC patients compared with the traditional targeted drug sunitinib [12]. Similarly, pembrolizumab combined with axitinib significantly improved patients’ objective response rate (ORR) [13]. However, the understanding of the application of adjuvant therapy after renal cancer surgery is still insufficient.

Despite some discrepancies in current research, more direct comparative studies are needed to evaluate the effectiveness of various agents. Researchers can utilize network meta-analysis to examine the efficacy of two different agents in studies with a placebo as the control group by incorporating direct and indirect comparisons. Hence, this research conducted an extensive assessment of the impacts of supplementary medications like sunitinib, sorafenib, and atezolizumab on survival measures such as DFS, overall survival (OS), and recurrence-free survival (RFS) in individuals following surgical removal of RCC via a meta-analysis involving multiple studies. The study also assessed the safety of these drugs to determine the best options for patients following RCC resection.

## Methods

The research was carried out following the PRISMA guidelines, and the meta-analysis protocol can be found on the PROSPERO website, the registration ID is CRD42023440272. The meta-analysis process strictly follows the checklist of the Preferred Reporting Items for Systematic Review and Meta-Analyses guidelines (Supplement table I).

### Search strategy

We searched multiple databases, such as CNKI (https://www.cnki.net/), WAN FANG DATA (https://www.wanfangdata.com.cn/), VIP (http://www.cqvip.com/), Web of Science (https://webofscience.clarivate.cn/), Cochrane Library (https://www.cochranelibrary.com/), PubMed (https://pubmed.ncbi.nlm.nih.gov/), Scopus (https://www.scopus.com/), and Embase (https://www.embase.com/), from inception to June 2, 2023, to find relevant studies. The search utilized the subsequent Mesh terms: (‘Kidney Tumors’ OR ‘Renal Tumors’ OR ‘Renal Malignancy’ OR ‘Kidney Malignancy’ OR ‘renal cell malignancy’ OR ‘renal cell tumor’ OR ‘kidney tumor’ OR ‘renal tumor’).

### Selection criteria

In order to establish inclusion, the researchers applied the following criteria: (1) individuals diagnosed with RCC; (2) previous nephrectomy and/or metastasectomy leading to complete remission; (3) patients who received adjuvant therapy after nephrectomy and/or metastasectomy; (4) documentation of DFS, OS, RFS, AEs with a grade of ≥ 3; and (5) RCTs. The exclusion criteria included the following: (1) non-experimental studies, correspondences, evaluations, or summaries from conferences; (2) studies with only one group; (3) studies involving animals or laboratory investigations; and (4) repeated literature publications.

### Extraction of data and evaluation of quality

Data from the included studies were independently extracted by two investigators (Guo LY and An T) using the Cochrane Risk of Bias 2.0 tool to evaluate the bias risk of each RCT. The senior reviewer (Huang ZX) resolved any inconsistencies. The collected data consisted of the primary author’s name, year of publication, patient count, medical condition, prescribed medications, the dosage of treatment, average duration of follow-up, severe AEs with a grade equal to or higher than 3, as well as the hazard ratios (HR) and corresponding 95% confidence intervals (CIs) for DFS, OS, and RFS.

### Data analysis

In order to assess the existence of incongruity, tests for both incongruity and congruity were performed. I^2^ was primarily used to assess the degree of heterogeneity. When there was no difference between the results (I^2^ ≤ 50%), the fixed effects model was used for meta-analysis; otherwise, the random effects model was used. Following the elimination of notable clinical variability, a random-effects approach was employed for the meta-analysis. To assess whether the statistical significance was achieved for DFS, OS, and RFS between any pair, a net-league table (referred to as a matrix in algebra) was employed. STATA 14.0 MP was utilized to perform traditional meta-analyses on AEs graded ≥ 3. This process generated Napierian logarithm odds ratios (lnOR) and their corresponding standard error (selnOR) for each individual study. Subsequently, the lnHR and selnHR values for DFS, OS, and RFS, along with the lnOR and selnOR values for AEs, were entered into R 4.3.1. The Netmeta package performed data processing, network data plots, and forest plots sequentially.

## Results

### The studies that were included had certain characteristics

**Fig. 1:**
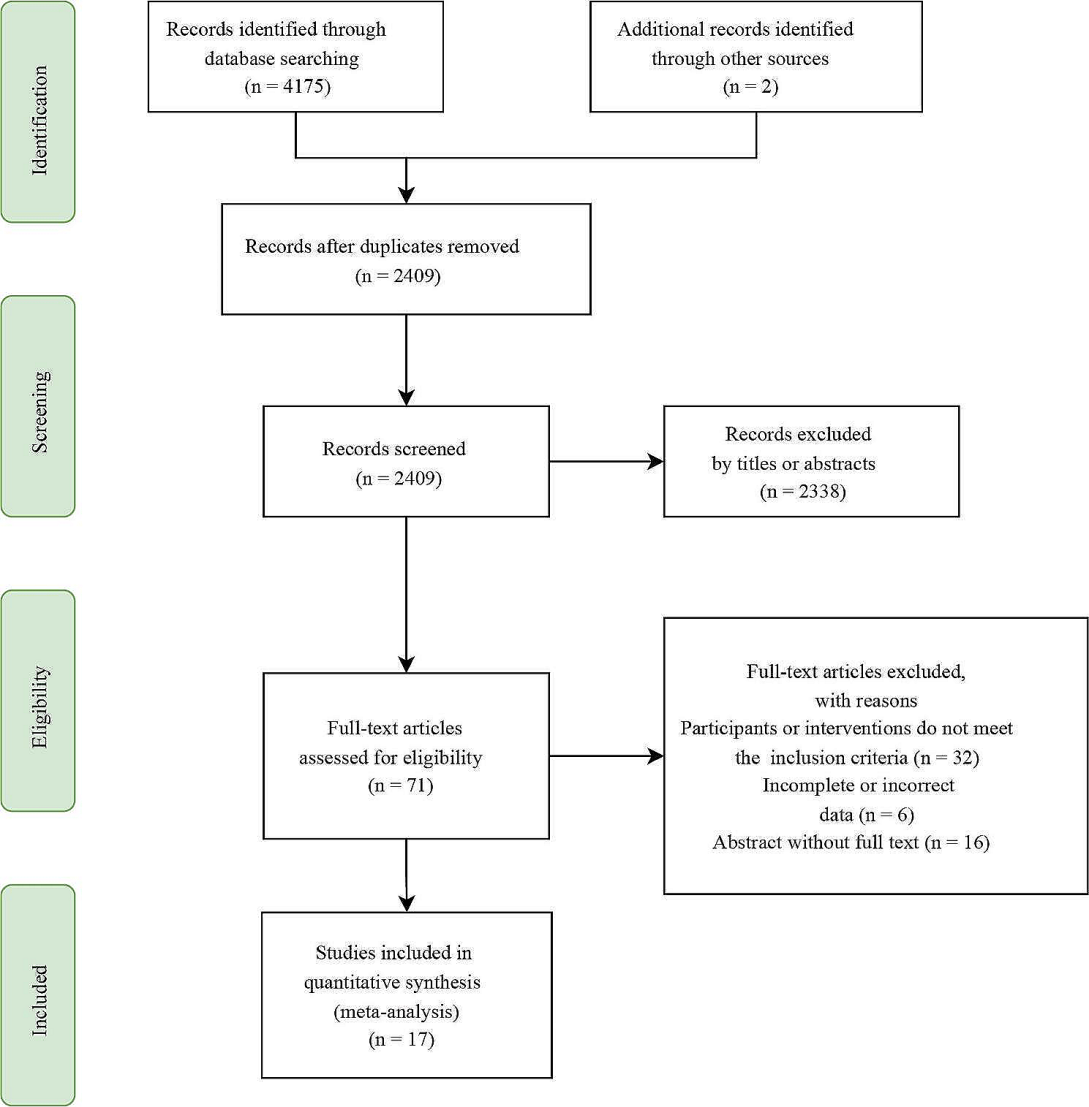
The flowchart shows the process of literature filtering

During the initial search period, we obtained a total of 4,177 publications published from 1977 to 2023. After removing duplicates and evaluating titles and abstracts, a total of 711 studies were considered suitable for a thorough examination, and eventually, 17 studies fulfilled our requirements (Fig. 1). Ultimately, all of the studies included 14,298 patients and compared 11 different treatments, specifically sunitinib, sorafenib, nivolumab in conjunction with ipilimumab, IL2 + IFN + 5FU, atezolizumab, pembrolizumab, brentuximab, tegafur in addition to uracil, thalidomide, and axitinib. We presented a comprehensive explanation of the included studies (Table 1). In these studies, all participants had fully recovered after undergoing a RCC nephrectomy, and the reported follow-up period varied from 24.1 to 112.9 months. Similarly, we tabulated the number of studies and patient samples included for different interventions (Table 2).

**Table 1:** Characteristics of studies included in the network meta-analysis

Study	Year of publication	Treatment	Sample size (T/C)	Median age	Gender (male/ female)	Follow up (month)	Therapeutic regimen	Outcomes
Robert J Motzer [14]	2023	nivolumab+ipilimumab	405	58	286/119	37	nivolumab (240 mg) every 2 weeks for 12 doses plus ipilimumab (1 mg/kg) every 6 weeks for 4 doses	DFS/AE
		placebo	411	57	294/117	37		
M. Aitchison [15]	2014	IL2 + IFN + 5FU	154	57	107/47	72	triple combination therapy (5-flurouracil, alpha-interferon (Roferon), interleukin-2(Proleukin)) no later than 12 weeks following surgery	DFS/OS
		observation	155	55	101/44	72		
Sumanta Kumar Pal [7]	2022	atezolizumab	390	61	287/103	60	atezolizumab (1200 mg intravenous) once every 3 weeks for 16 cycles or 1 year	DFS/AE
		placebo	388	60	278/110	60		
Robert J. Motzer [9]	2021	pazopanib	769	58	537/232	76	pazopanib or placebo for 1 year	OS
		placebo	769	59	554/215	76		
Choueiri, Toni K [16]	2021	pembrolizumab	496	60	347/149	24.1	pembrolizumab(200 mg) once every 3 weeks for up to 17cycles (approximately 1 year)	DFS/AE
		placebo	498	60	359/139	24.1		
Tim Eisen, [17]	2020	sorafenib	639	58	458/181	78	sorafenib 400 mg twice per day orally	DFS/OS/AE
		sorafenib + placebo	642	58	452/190	78		
		placebo	430	58	306/124	78		
Robert J. Motzer [18]	2018	sunitinib	309	57	222/87	60	sunitinib or placebo for nine cycles (_1 year)	DFS/OS/AE
		placebo	306	58	230/76	78		
Naomi B Haas [5]	2016	sunitinib	647	56	429/218	60	sunitinib 50 mg per day orally, sorafenib 400 mg twice per day orally for 54 weeks	DFS/OS/AE
		sorafenib	649	55	437/212	60		
		placebo	647	57	443/204	60		
J Atzpodien [19]	2005	IL2 + IFN + 5FU	135	59	97/38	51.6	subcutaneous interleukin-2, interferon-alpha2a, and intravenous 5-fluorouracil for 8 weeks	RFS/OS
		observation	68	60	54/14	51.6		
Karim Chamie [20]	2017	girentuximab	433	58	276/157	54	girentuximab, 50 mg (week 1), followed by weekly intravenous infusions of girentuximab, 20 mg (weeks 2–24)	DFS/OS/AE
		placebo	431	58	298/133	54		
Giuseppe Procopio [21]	2019	sorafenib	32	65	20/12	38	sorafenib (standard dose 400 mg twice daily) for 52 wk	RFS/OS/AE
		observation	36	59	45/80	38		
Thomas Powles [22]	2021	pembrolizumab	496	60	347/149	30.1	pembrolizumab 200 mg intravenously every 3 weeks for up to 17 cycles	DFS/OS/AE
		Placebo	498	60	359/139	30.1		
Seiji Naito [23]	1997	tegafur + uracil	33	-	22/11	112.9	tegafur and uracil (300 to 600 mg as tegafur) every day for 2 years	RFS/AE
		observation	33	-	22/11	112.9		
A. Mennitto [24]	2021	sorafenib	32	65	20/12	42	sorafenib (standard dose 400 mg twice daily) for 52 wk	RFS
		observation	36	59	45/80	42		
Robert J. Motze [25]	2017	pazopanib600mg	571	58	398/173	47.9	pazopanib 800 mg once daily as the starting dose, then reduced to 600 mg once daily	DFS/OS/AE
		placebo	564	58	400/164			
		pazopanib800mg	198	56	139/59	47.9		
		placebo	205	60	154/51	47.9		
Naomi B. Haas [26]	2017	sunitinib	358	58	243/115	60	sunitinib (50 mg), sorafenib (800 mg) for 1 year	DFS/OS
		sorafenib	355	57	248/107	60		
		placebo	356	58	254/102	60		
M. Gross-Goupil [11]	2018	axitinib	363	58	280/83	31	axitinib 5 mg twice-daily oral	DFS/AE
		placebo	361	58	250/11	31		

DFS, disease-free survival; OS, overall survival; RFS, recurrence-free survival; AE, adverse event

**Table 2:** The number of studies and patient samples included for different interventions

Treatment	Study	Sample size
nivolumab + ipilimumab	1	405
IL2 + IFN + 5FU	2	289
atezolizumab	1	390
pazopanib	2	1538
pembrolizumab	2	992
sorafenib	5	2349
sunitinib	3	1314
girentuximab	1	433
tegafur + uracil	1	33
axitinib	1	363

### Potential for bias in research

**Fig. 2:**
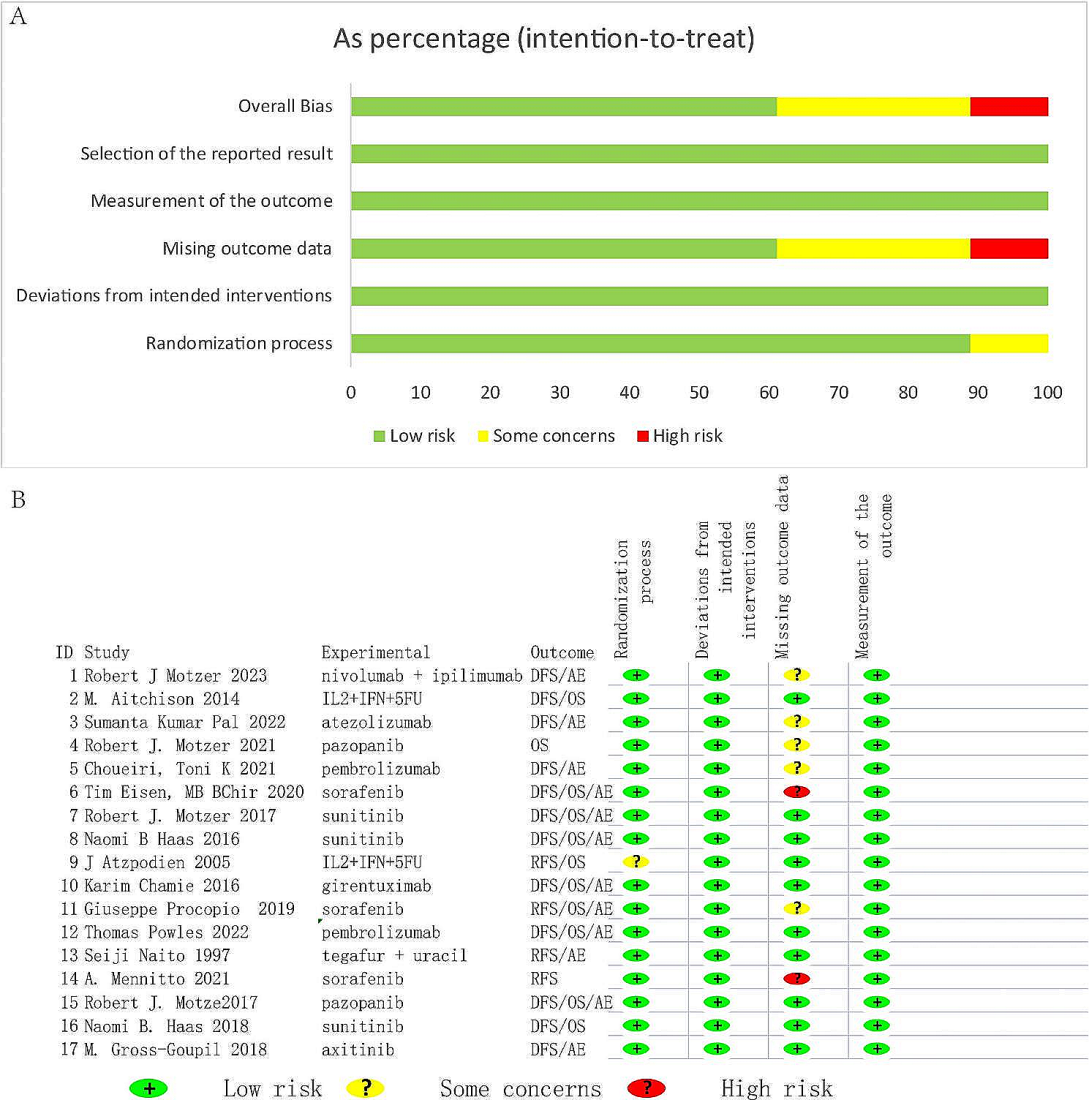
Risk of bias for all randomized controlled trials included in this study. (**A**) Bar chart of bias; (**B**) Risk of bias summary. DFS, Disease-free survival; OS, Overall survival; RFS, Recurrence-free survival; AEs, adverse events; IL2 + IFN + 5FU, interleukin-2 + interferon-alpha2a + 5-fluorouracil

Out of all the research conducted, 6 were categorized as open-label. The method of randomization was not provided in 1 article, and 7 articles mentioned withdrawals of over 20% during the follow-up period. The details of the risk of bias assessment for each study are presented in Fig. 2 (Fig. 2A and B).

### Survival analysis of DFS, OS, and RFS

**Fig. 3:**
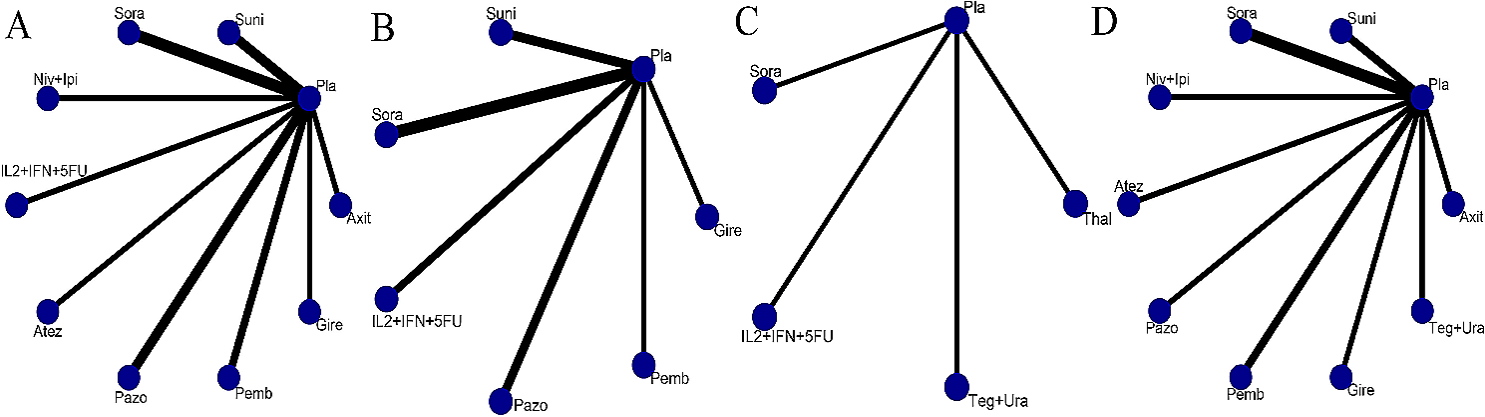
Network diagrams of outcome indicators. (**A**) Disease-free survival (DFS); (**B**) Overall survival (OS); (**C**) Recurrence-free survival (RFS); (**D**) adverse events (AEs). Pla, placebo; Suni, sunitinib; Sora, sorafenib; Niv + Ipi, nivolumab + ipilimumab; IL2 + IFN + 5FU, interleukin-2 + interferon-alpha2a + 5-fluorouracil; Atez, atezolizumab; Pazo, pazopanib; Pemb, pembrolizumab; Gire, girentuximab; Teg + Ura, tegafur + uracil; Thal, thalidomide; Axit, axitinib

**Fig. 4:**
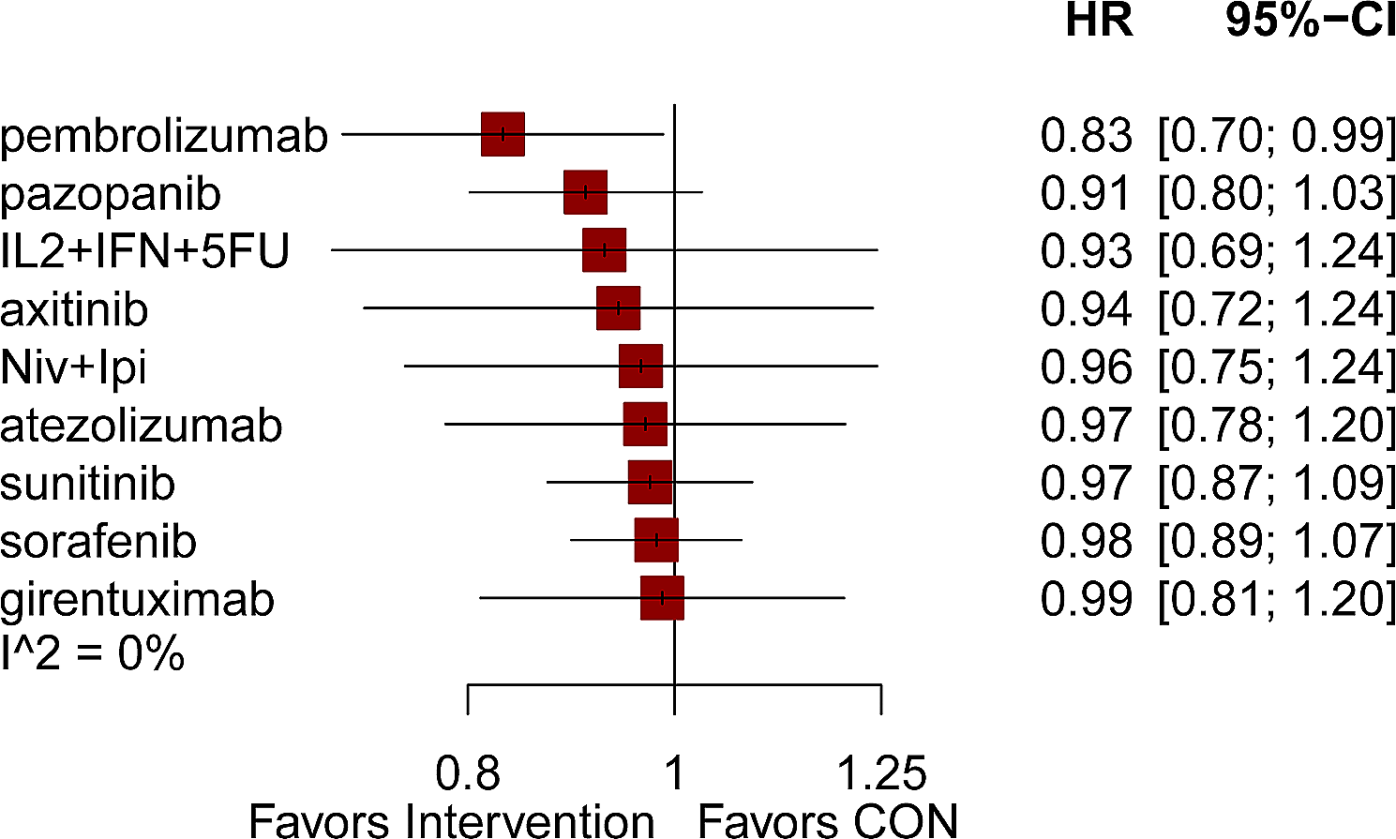
Effects of different interventions on disease-free survival

The comparative relationship between different interventions was shown in a network diagram (Fig. 3A-D). Among the 17 articles, 12 provided information on the HRs concerning DFS [5, 7, 11, 14–18, 20, 22, 25, 26]. We compared the 10 interventions included in the network diagram, both directly and indirectly. The most used agent was sorafenib, the most common comparison was between sorafenib and placebo. The intervention measures that exhibited notable distinctions compared to the placebo were pembrolizumab (HR = 0.83, 95%CI 0.70 to 0.99). Adjuvant treatments such as pazopanib, interleukin-2 + interferon-alpha2a + 5-fluorouracil (IL2 + IFN + 5FU), axitinib, nivolumab plus ipilimumab, atezolizumab, sunitinib, sorafenib, and girentuximab had no effect on DFS(HRs from 0.91 to 0.99)(Fig. 4). After conducting a network comparison, a grand total of 45 pairwise comparisons were obtained. The findings indicated that there was no notable disparity observed in DFS among these treatments. Table displays the precise outcomes (Table 3).

**Table 3:** The NMA presents the impact of each intervention for disease-free survival

Placebo	1.03 (0.92,1.15)	1.02 (0.93,1.12)	1.04 (0.80,1.34)	1.08 (0.80,1.45)	1.03 (0.83,1.28)	1.10 (0.97,1.25)	1.20 (1.01,1.43)	1.01 (0.83,1.23)	1.06 (0.81,1.40)
1.03 (0.92,1.15)	sunitinib	NA	NA	NA	NA	NA	NA	NA	NA
1.02 (0.93,1.12)	0.99 (0.86,1.15)	sorafenib	NA	NA	NA	NA	NA	NA	NA
1.04 (0.80,1.34)	1.01 (0.76,1.33)	1.02 (0.78,1.33)	nivolumab + ipilimumab	NA	NA	NA	NA	NA	NA
1.08 (0.80,1.45)	1.05 (0.77,1.44)	1.06 (0.78,1.44)	1.04 (0.71,1.54)	IL2 + IFN + 5FU	NA	NA	NA	NA	NA
1.03 (0.83,1.28)	1.00 (0.79,1.28)	1.01 (0.80,1.28)	1.00 (0.71,1.39)	0.96 (0.66,1.38)	atezolizumab	NA	NA	NA	NA
1.10 (0.97,1.25)	1.07 (0.91,1.27)	1.08 (0.92,1.26)	1.06 (0.80,1.41)	1.02 (0.74,1.40)	1.07 (0.83,1.37)	pazopanib	NA	NA	NA
1.20 (1.01,1.43)	1.17 (0.95,1.44)	1.18 (0.97,1.44)	1.16 (0.85,1.58)	1.12 (0.79,1.57)	1.17 (0.88,1.54)	1.09 (0.88,1.35)	pembrolizumab	NA	NA
1.01 (0.83,1.23)	0.99 (0.79,1.24)	0.99 (0.80,1.23)	0.98 (0.71,1.35)	0.94 (0.66,1.34)	0.98 (0.73,1.31)	0.92 (0.73,1.16)	0.84 (0.65,1.09)	girentuximab	NA
1.06 (0.81,1.40)	1.03 (0.77,1.39)	1.04 (0.78,1.39)	1.02 (0.70,1.49)	0.98 (0.66,1.47)	1.03 (0.73,1.46)	0.97 (0.71,1.30)	0.88 (0.64,1.22)	1.05 (0.75,1.47)	axitinib

**Fig. 5:**
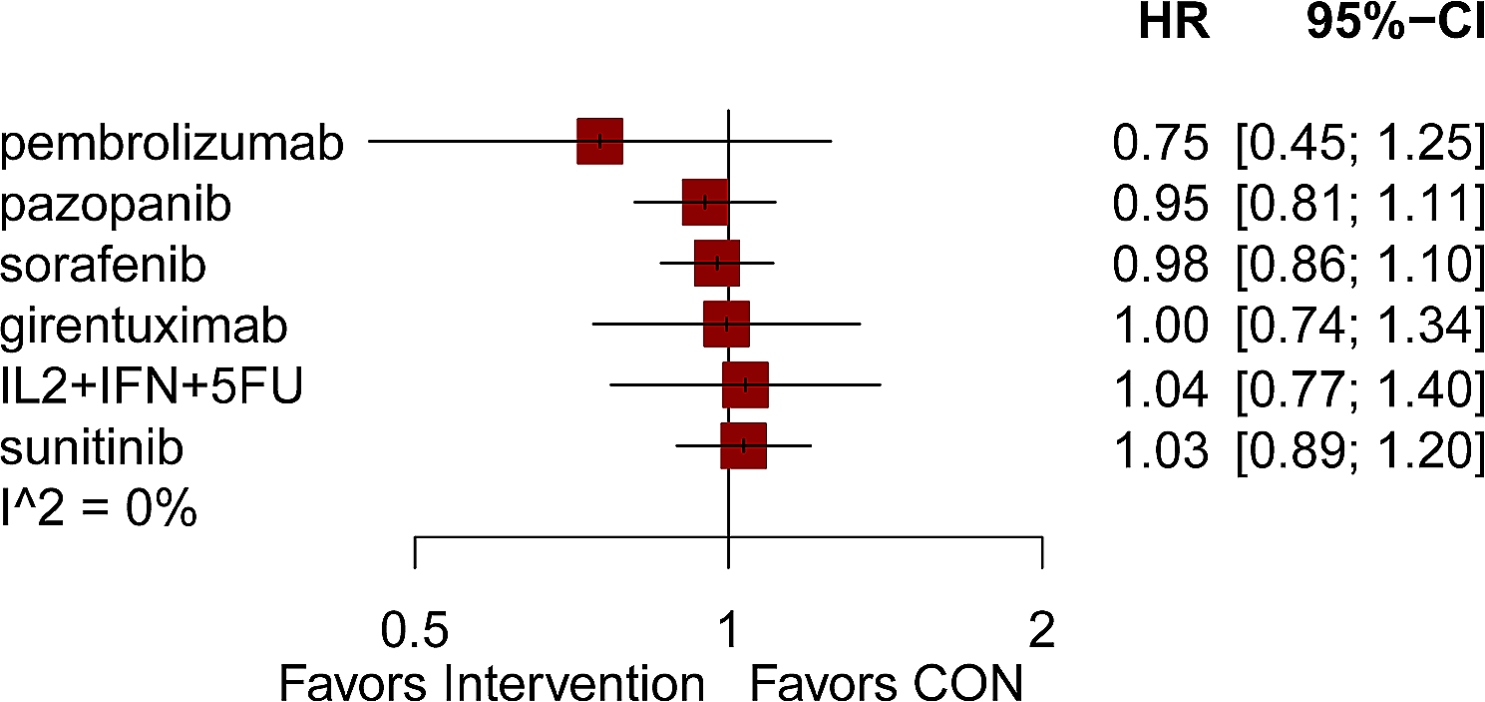
The impact of various interventions on overall survival

11 of the 17 articles provided OS findings [5, 9, 15, 17–22, 25, 26]. Figure 3 presented a network graph that included 7 interventions, which were compared directly and indirectly (Fig. 3B). The thickness of the lines in the network graph represents the number of studies included, from which we can see that the most commonly used drug was sorafenib, and the most common comparison was between sorafenib and placebo. Adjuvant therapies including pembrolizumab, pazopanib,sorafenib, girentuximab, IL2 + IFN + 5FU and sunitinib could not influence the OS(HRs from 0.75 to 1.04) (Fig. 5). After conducting a network analysis, a grand total of 21 pairwise comparisons were obtained. The findings indicated no notable distinction was observed in OS among these treatments (Table 4).

**Table 4:** The NMA presents the impact of each intervention for overall survival

Placebo	0.97 (0.83,1.12)	1.03 (0.91,1.16)	0.96 (0.72,1.30)	1.05 (0.90,1.23)	1.33 (0.80,2.21)	1.00 (0.75,1.35)
0.97 (0.83,1.12)	sunitinib	NA	NA	NA	NA	NA
1.03 (0.91,1.16)	1.06 (0.87,1.29)	sorafenib	NA	NA	NA	NA
0.96 (0.72,1.30)	1.00 (0.72,1.39)	0.94 (0.68,1.30)	IL2 + IFN + 5FU	NA	NA	NA
1.05 (0.90,1.23)	1.09 (0.88,1.35)	1.03 (0.84,1.25)	1.09 (0.78,1.53)	pazopanib	NA	NA
1.33 (0.80,2.21)	1.37 (0.81,2.33)	1.30 (0.77,2.19)	1.38 (0.76,2.49)	1.26 (0.74,2.15)	pembrolizumab	NA
1.00 (0.75,1.35)	1.04 (0.75,1.44)	0.98 (0.71,1.35)	1.04 (0.69,1.58)	0.95 (0.68,1.33)	0.76 (0.42,1.36)	girentuximab

**Fig. 6:**
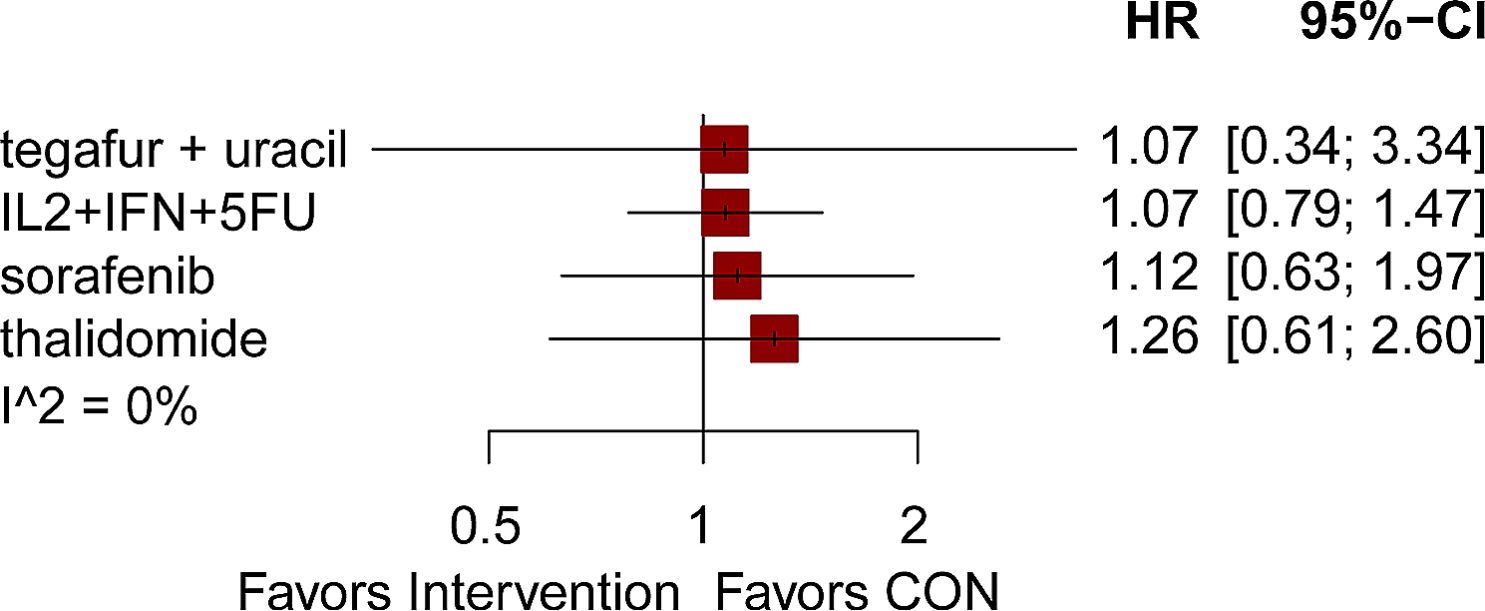
Effects of different interventions on recurrence-free survival

Of the 17 articles, 4 provided information on RFS outcomes [19, 21, 23, 24]. Figure 3 displays a network graph comparing the 4 interventions, both directly and indirectly (Fig. 3C). In terms of RFS, the frequency of the four drugs was the same. Adjuvant therapies including tegafur plus uracil, IL2 + IFN + 5FU, sorafenib, and thalidomide could not influence the RFS(HRs from 1.07 to 1.26) (Fig. 6). After conducting a network analysis, a grand total of 10 pairwise comparisons were obtained. The findings indicated that there was no notable distinction observed in RFS among these interventions (Table 5).

**Table 5:** The NMA presents the impact of each intervention for recurrence-free survival

placebo	0.90 (0.51,1.58)	0.93 (0.68,1.27)	0.93 (0.30,2.91)	0.79 (0.38,1.64)
0.89 (0.51,1.58)	sorafenib	NA	NA	NA
0.93 (0.68,1.27)	1.04 (0.54,1.99)	IL2 + IFN + 5FU	NA	NA
0.93 (0.30,2.91)	1.04 (0.29,3.72)	1.00 (0.31,3.26)	tegafur + uracil	NA
0.79 (0.38,1.64)	0.89 (0.35,2.23)	0.85 (0.39,1.88)	0.85 (0.22,3.27)	thalidomide

### Analysis of AEs (grade ≥ 3)

**Fig. 7:**
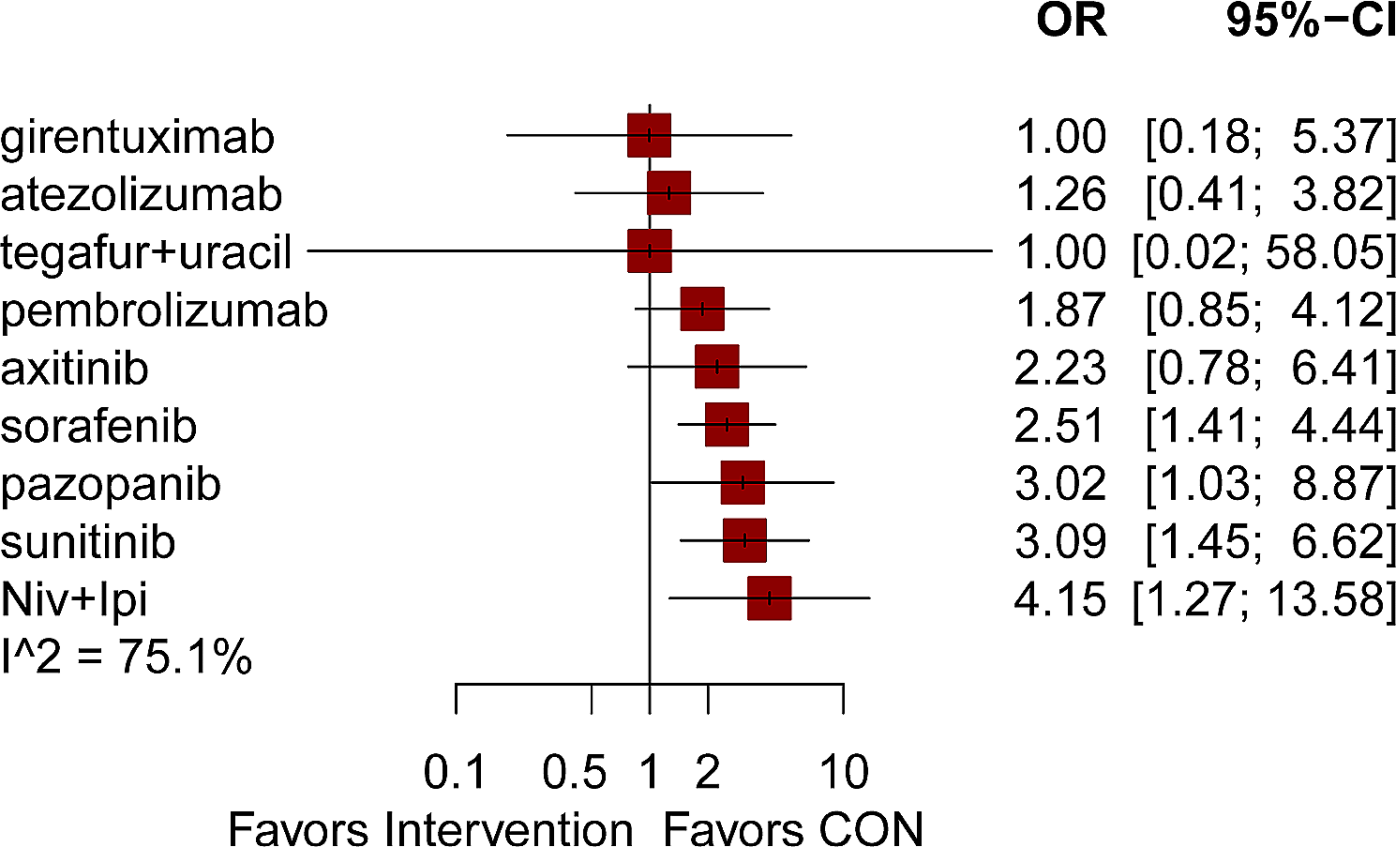
Effects of different interventions on adverse events

Out of the 17 articles, AEs of grade ≥ 3 were reported in 12 of them [5, 7, 11, 14, 16–18, 20–23, 25]. A network graph in Fig. 3 also displayed comparisons between 10 interventions (Fig. 3D). The most used drug in this part of the study was sorafenib, with the most compared between sorafenib and placebo. Most drugs were found to be more toxic than placebos. The intervention measures that exhibited significant variations in comparison to placebo. Adjuvant treatments including sorafenib, pazopanib, sunitinib, and nivolumab plus ipilimumab suggested a higher likelihood of AEs (ORs 2.51 to 4.15). The remaining adjuvant treatments, including girentuximab, atezolizumab, tegafur plus uracil, pembrolizumab, and axitinib, did not affect AEs (ORs 1.0 to 2.23) (Fig. 7). After conducting a network comparison, a grand total of 45 pairwise comparisons were obtained. The findings indicated no notable distinction was observed in the AEs among these treatments (Table 6).

**Table 6:** The NMA presents the impact of each intervention for adverse events

Placebo	0.32 (0.15,0.69)	0.40 (0.22,0.71)	0.24 (0.07,0.79)	0.80 (0.26,2.42)	0.33 (0.11,0.97)	0.54 (0.24,1.18)	1.00 (0.19,5.42)	1.00 (0.02,58.05)	0.45 (0.16,1.29)
0.32 (0.15,0.69)	sunitinib	NA	NA	NA	NA	NA	NA	NA	NA
0.40 (0.22,0.71)	1.23 (0.48,3.20)	sorafenib	NA	NA	NA	NA	NA	NA	NA
0.24 (0.07,0.79)	0.75 (0.18,3.05)	0.60 (0.16,2.25)	nivolumab + ipilimumab	NA	NA	NA	NA	NA	NA
0.80 (0.26,2.42)	2.46 (0.64,9.47)	1.99 (0.57,6.96)	3.30 (0.65,16.77)	atezolizumab	NA	NA	NA	NA	NA
0.33 (0.11,0.97)	1.02 (0.27,3.83)	0.83 (0.24,2.81)	1.37 (0.28,6.82)	0.42 (0.09,1.96)	pazopanib	NA	NA	NA	NA
0.54 (0.24,1.18)	1.66 (0.55,4.96)	1.34 (0.51,3.56)	2.22 (0.53,9.24)	0.67 (0.17,2.63)	1.62 (0.42,6.15)	pembrolizumab	NA	NA	NA
1.00 (0.19,5.42)	3.11 (0.49,19.74)	2.52 (0.42,14.92)	4.17 (0.53,32.72)	1.26 (0.17,9.50)	3.03 (0.41,22.42)	1.88 (0.29,12.06)	girentuximab	NA	NA
1.00 (0.02,58.05)	3.09 (0.05,192.80)	2.51 (0.04,151.46)	4.15 (0.06,285.53)	1.26 (0.02,84.73)	3.02 (0.05,201.79)	1.87 (0.03,117.00)	1.00 (0.01,80.82)	tegafur + uracil	NA
0.45 (0.16,1.29)	1.39 (0.38,5.10)	1.12 (0.34,3.74)	1.86 (0.38,9.11)	0.56 (0.12,2.61)	1.36 (0.30,6.13)	0.84 (0.22,3.13)	0.45 (0.06,3.26)	0.45 (0.01,29.81)	axitinib

## Discussion

As more and more clinical studies explore additional treatments for removing RCC, there is a simultaneous increase in clinical reports discussing various therapies [27]. However, we need to further clarify which adjuvant therapy is best for RCC. Conventional meta-analyses, limited to pairwise comparisons, might need more methodological assistance in determining the most efficacious intervention. Network meta-analysis allows for the comparison of various interventions [28]. Hence, this research utilized network meta-analysis for the initial occasion to evaluate the effectiveness and security of different supplementary therapies following surgical removal of RCC.

Regarding DFS, pembrolizumab was the only adjuvant drug that exhibited a noteworthy enhancement in comparison to a placebo (HR = 0.83, 95% CI 0.70 to 0.99); the remaining adjuvant drugs did not display any significant impact. Furthermore, given the inconclusive outcomes from previous sunitinib trials, our investigation determined sunitinib’s HR to be 0.97, with a 95% CI ranging from 0.87 to 1.09. Considering these results, we exercise prudence when considering the utilization of sunitinib to improve DFS in individuals who have undergone surgery for RCC. Previous research has not witnessed any enhancement in OS and RFS when employing supplementary medications. In our study, we reached a consistent finding that additional medications did not significantly improve postoperative OS and RFS results for individuals diagnosed with RCC when compared to a placebo. Due to the extended survival period following surgery in individuals with RCC, certain clinical studies might not have documented significant occurrences of OS. Hence, it might be essential to create novel medications and conduct more clinical experiments to validate the possible enhancement in OS and RFS among individuals who have undergone surgery for RCC.

In terms of AEs with a grade of ≥ 3, the included interventions of sorafenib, pazopanib, sunitinib, and nivolumab plus ipilimumab showed a higher incidence of adverse reactions compared to a placebo. The safety comparison between different interventions did not demonstrate a significant difference. First-line treatment should not include sorafenib, pazopanib, sunitinib, or nivolumab plus ipilimumab as they were determined to have no effect on improving DFS, OS, and RFS in patients who underwent RCC resection.

These findings indicate that among the existing drugs, pembrolizumab improves DFS in patients following RCC resection, while other drugs do not significantly enhance survival. Checkpoint inhibitors and TKIs are two different anticancer drugs with different mechanisms of action and effects in the treatment of tumors. Checkpoint inhibitors activate the patient’s own immune system to attack tumor cells by disarming immune checkpoints such as PD-1 and CTLA-4 [29]. This mechanism of immune activation can lead to a durable immune response and show significant therapeutic effects in multiple tumor types. Checkpoint inhibitors can trigger a long-lasting immune response, meaning that the immune system is able to recognize and attack tumor cells even after drug treatment is stopped. In contrast, the efficacy of TKIs is often associated with the presence and continued use of the drug. Checkpoint inhibitors can activate multiple types of immune cells, including T cells, B cells, and natural killer cells, leading to a more comprehensive anti-tumor immune response. TKIs inhibit the growth and spread of tumor cells mainly by interfering with signal transduction pathways. Tumor cells often evade the effects of TKIs through a variety of mechanisms, such as the development of drug-resistant mutations and the activation of alternative signaling pathways. However, checkpoint inhibitors, by boosting the activity of the immune system, can respond to situations in which tumor cells escape, thereby reducing the development of drug resistance.

RCC patients have significantly elevated levels of VEGF-A compared to patients with other types of cancer, indicating that RCC is a tumor that is rich in blood vessels [30]. Moreover, TKIs can improve vascularization, directly or indirectly increasing immune infiltration. Significant results were shown in the treatment of metastatic RCC with immune checkpoint inhibitors in the year 2016 [27]. Hence, the collective utilization of various medications exhibits a more potent ability to combat tumors, and the integration of additional supportive treatments alongside pembrolizumab presents a novel method to prolong the survival of patients who have undergone surgery for RCC. However, treatment-related toxicity must also be considered when using multiple adjunctive therapies in combination, as immune checkpoint inhibitors are susceptible to immune-related adverse events, while TKIs have chronic toxicity. While combination drug therapy may have more significant toxicity than single therapy, treatment plans that control toxicity within an acceptable range still have significant potential for application.

## Conclusion

The network meta-analysis results showed that pembrolizumab was successful in enhancing DFS in patients who underwent surgery for RCC compared to a placebo. Additionally, the treatment did not lead to any significant toxicity. The assessment is a valuable guide for postoperative adjuvant therapy in individuals diagnosed with RCC.

### Electronic supplementary material

Below is the link to the electronic supplementary material.

Supplementary Material 1

## Data Availability

The data that support the findings of this study are available from the corresponding author upon reasonable request.
